# A longitudinal analysis on determinants of problem drinking among Korean women: focusing on a gender perspective

**DOI:** 10.1186/s13011-022-00481-3

**Published:** 2022-07-15

**Authors:** Soo-Bi Lee, Hyung-Joo Park, Myeong-Sook Yoon

**Affiliations:** Department of Social Welfare, Jeonbuk National University, 567 Baekje-daero, Deokjin-gu, Jeonju-si, Jeollabuk-do 54896 Republic of Korea

**Keywords:** Female drinking, Change patterns, Problem drinking predictors, Gender specific experiences, Latent class growth analysis

## Abstract

**Background:**

In recent years, female drinking has been on the rise worldwide, and this trend can be observed in Korea as well. Accordingly, this study aimed to examine the heterogeneous longitudinal changes in drinking patterns among Korean women, while also exploring the determinants of these changes. In particular, the study identified the gender perspective-related determinants of the classified patterns of problem drinking.

**Methods:**

Data on 4615 adult women who participated in the Korea Welfare Panel Study (KOWEPS) for 3 years (2018–2020) were analyzed longitudinally using SPSS Statistics 22.0 and M-plus 7.0. The changes in female drinking patterns were analyzed using latent class growth analysis. Subsequently, multinomial logistic regression analysis was performed to identify the predictive factors affecting the changes in drinking patterns.

**Results:**

Latent class analysis yielded three classes: “low problem drinking/decreased,” “moderate problem drinking/maintained,” and “high problem drinking/increased.” Of the participants, 80.4% were in the first class, 14.5% in the second, and 5.1% in the third. After controlling for sociodemographic and psychosocial factors, we found: i) domestic violence, work–family balance stress, and gender role perception were not statistically significant for the “moderate problem drinking/maintained” class; lower levels of depression (odds ratio; OR = .750, *p* < .05) and higher levels of satisfaction with social relationships (OR = 1.257, *p* < .05) increased the probability of belonging to the “moderate problem drinking/maintained” group compared to the low problem drinking/decreased class; ii) in the “high problem drinking/increased” class, relative to the low problem drinking/decreased class, experience of domestic violence (OR = 1.857, *p* < .05), work–family balance stress (OR = 1.309, *p* < .05), and gender role perception (OR = .705, *p* < .05) were significant predictors of drinking behavior.

**Conclusions:**

Problem drinking in Korean women demonstrated heterogeneous patterns of change, with gender-specific factors being the main predictors of this change. Therefore, this study developed a strategy for reducing the harmful effects of female drinking, which considers the characteristics of the changes in women’s drinking patterns as well as factors from the gender perspective.

## Background

While there has been a general decline in alcohol consumption in many countries in recent years, an increasing trend of hazardous drinking behaviors, such as high-risk drinking and binge drinking, has been noted among women and youth [1, 2]. Traditionally, the absolute incidence of alcohol consumption or problem drinking has been markedly higher among men. However, recent studies indicate that the gender gap in various indices of alcohol use is narrowing by age group [3, 4]. This evidence suggests that negative social and cultural views of female drinking are declining, and gender role norms are changing.

This rising rate of female drinking is evident in Korea as well. While the rate of male drinking dropped slightly from 21.4 to 20.8% in 2019, female drinking rates rose sharply from 5.4 to 8.4% during the same period [5]. Consequently, there has been mounting interest in female drinking behaviors in Korean society.

The discussion over problem drinking has been primarily focused on men. Recently, however, there has been an increased interest in the gender differences in the prevalence, clinical characteristics, biological vulnerability, and course of treatment [1, 6]. As women are more vulnerable to the physical effects of alcohol compared to men, they are at greater risk of health problems, sexual violence, binge drinking, and problem drinking [7–9]. Female drinking is viewed in a more negative light than male drinking in Korean culture, and this hinders early or timely intervention for drinking problems [10], resulting in a quicker progression to alcohol dependence [11]. Further, excessive drinking is considered a serious public health problem due to the adverse effects of heavy drinking during pregnancy and childbirth in women of childbearing age [1, 12–14]. From this perspective, some researchers have argued that female drinking must be treated as a different social phenomenon as opposed to male drinking [15], and measures must be devised accordingly [15–17]. However, research approaches to female drinking have remained fragmentary.

Studies thus far have noted differences between men and women in terms of the biological impact of alcohol consumption and drinking predictors [13]. For example, men often engage in social drinking, but women tend to drink to relieve negative emotions. In this context, many studies have pointed to psychosocial factors—such as stress and coping mechanisms [18, 19], low self-esteem, depression, social support, and social networks [20–22]—as predictors of problem drinking in women. Furthermore, sociodemographic factors such as age, education level, marital status, income, economic activity status [9, 16, 23–25], one-person households [26], and relationship-related factors [27, 28] have also been mooted as predictors of female drinking.

Meanwhile, some scholars have argued that the perspective of gender is necessary to pinpoint the underlying causes of increased drinking among women accurately. This necessity is accentuated considering compositional and social contextual characteristics such as gender roles and norms that have changed over time, [3, 9, 16]. In other words, they assert that drinking behavior and its levels of harm would appear differently in women, depending on the interaction of various factors such as their social position and role, the gender norms that influence their values and beliefs associated with drinking, the industrial environment related to the availability of alcohol, and socio-cultural environmental characteristics as well as their physiological characteristics regarding alcohol [17, 29–31].

However, previous studies have a limitation in that they are mainly described based on demographic variables [17, 32, 33]. Moreover, they focus on women’s vulnerable psycho-emotional characteristics, thereby limiting one’s understanding of the fundamental reasons behind their increased alcohol consumption from a social contextual perspective. Therefore, identifying more fundamental causes behind female drinking is essential, specifically in terms of the effects of gender-related experiences—which are rooted in social structures and family systems—on women’s drinking.

Traditionally, it has been accepted that culturally defined gender roles shape male and female drinking patterns [34, 35]. Some studies report that conservative and traditional patriarchal gender norms that stress women’s involvement in parenting and household chores are negatively associated with female drinking [29, 36, 37]. Domestic violence has been identified as another gender-related factor, with female victims found to engage in hazardous drinking for various reasons, including escaping their trauma [38–42]. Therefore, it has been speculated that gender factors, such as women’s perceived gender roles and experience of domestic violence, can help predict problem drinking in women.

Problem drinking involves cumulative and continuous drinking, which has harmful consequences. As drinking is a type of social behavior [43], for an accurate understanding of the issue, problem drinking among women must be understood in the social context—as opposed to viewing it as a phenomenon specific to a single point in time. However, many studies have adopted a cross-sectional design, and some longitudinal studies that have applied the latent growth model have assumed that the entire population has experienced homogeneous changes. Hence, previously reported data have failed to shed light on the different patterns of changes in drinking within a given population.

Therefore, this study aims to identify the types of longitudinal changes in female drinking behaviors and explores their characteristics. It also seeks to investigate the effects of gender-related experiences of women arising from the social structure—domestic violence, work–family balance stress, and perceived gender roles—on the types of longitudinal changes in their drinking. Based on our findings, we present clinical and policy directions to reduce the negative effects of problem drinking behaviors in women.

## Methods

### Data & participants

We used secondary sources of data of 4615 adult women collected during the 13th–15th (2018–2020) Korea Welfare Panel Study (KOWEPS) for our analysis. The KOWEPS, conducted by the Korean Institute of Social and Health Affairs in conjunction with the Social Welfare Research Institute of Seoul National University, is an ongoing National Approval Statistics (No. 33109) of nationally representative Korean households. The purpose of this panel survey was to dynamically identify the socio-economic characteristics of the population and the multidimensional changes in living situation amid social change, and to investigate the welfare needs of each population group, ultimately contributing to the formation of welfare policies. Surveys were conducted by trained individuals in the form of face-to-face interviews at the participants’ homes using structured questionnaires. The survey is repeated annually, and proportional systematic stratified cluster sampling was used to select participants. Further details of the sampling design, methods and data sets can be found on the relevant website (https://www.koweps.re.kr). Especially, the KOWEPS provides representative and reliable data for longitudinal research [44]. According to the secondary data (raw data) used, there were 7516 adult women in the first year (2018), 7236 in the second year, and 6727 in the third year. 4615 women who responded to the survey all 3 years were used as the subjects of the analysis, excluding cases with missing utilization variables. This study was approved by the relevant Institutional Review Board according to the Declaration of Helsinki [irb 21–016-00].

### Measures

#### Alcohol use disorder identification test

The dependent variable in this study was problem drinking, which was measured using the Alcohol Use Disorder Identification Test (AUDIT)—a 10-item screening test developed by the World Health Organization. Each item is rated on a five-point Likert scale, with the total score ranging from 0 to 40. This scale is interpreted based on a cut-off, with an AUDIT score of 6 or higher indicating hazardous alcohol consumption in women, and a score of 10 or higher indicating alcohol use disorder. However, the total score is sometimes interpreted based on a continuum of scores. A higher score indicated a higher level of problem drinking and a higher risk of engaging in hazardous drinking behaviors.

#### Experience of domestic violence

Experience of domestic violence was measured using three items: “insulting and malicious words,” “threat of physical violence,” and “direct infliction of physical violence.” The items were rated on a scale of 0 (“never”) to 3 (“more than 6 times”). The total score was determined by summing the individual scores. A higher score indicates more experience of domestic violence.

#### Challenges in work–family balance

We used eight modified items [44] from the Family and Changing Gender Roles I–III modules developed by the International Social Survey Programme (ISSP). The eight items were broadly divided into three constructs. The overall scale or each subscale can be used by researchers, depending on their views and objectives, after making the necessary modifications [45, 46]. “Challenges in work–family balance” refer to the degree of difficulty in achieving work–family balance and were measured using three items: “Family life gives me stress,” “It is difficult to fulfill my family responsibilities,” and “It is difficult to concentrate at work because of my family responsibilities.” The items were rated on a five-point scale ranging from 1 to 5 (1 = strongly disagree and 5 = strongly agree), with a higher score indicating greater challenges to achieving work–family balance.

#### Gender role values

Gender role values were measured using two items from the ISSP instrument [47]: “Men’s duty is to make money, and women’s duty is to tend to the home and the family’s needs” and “Both men and women must contribute to the household income (reverse-coding).” The two scores were added to obtain the total score, with a higher score indicating a more traditional gender role, reflecting a gender-discriminatory attitude [45], and a lower score indicating a more flexible view regarding gender roles [48].

#### Depression

Depression was measured using the Center for Epidemiologic Studies Depression Scale (CES-D). The scale, which was developed for easy measurement of depression in the general population, is widely used in research, both in Korea and other countries. The 11-item scale (with a four-point rating scale ranging from 0 to 3) is a self-report questionnaire for measuring individuals’ psychological attitudes and behaviors; generally, a higher total score indicates more severe depression. For this study, we used the total score as a continuous variable.

#### Satisfaction with social relationships

The degree of satisfaction with social relationships was measured using a five-point Likert scale, with a higher score indicating greater satisfaction with social relationships.

#### Sociodemographic factors

Sociodemographic factors include age, religion, education level, employment status (economic activity status), presence of a spouse, and (equivalized) income. For this study, age and education level were used as continuous variables, and religion, presence of a spouse, and employment status were used as dummy variables (no = 0, yes = 1). Equivalized income was analyzed as a natural logarithm (ln) to ensure the normality of the data.

### Statistical analysis

The data were analyzed using SPSS Statistics 22.0 and M-plus 8.0 software. First, the general characteristics and main variables were analyzed using frequencies and descriptive statistics. Second, the patterns of changes in drinking level among women were analyzed using latent class growth analysis (LCGA). Latent growth modeling—the primary method of analysis chosen by existing longitudinal research—assumes that the study population undergoes an equal pattern of change. Therefore, it cannot detect the different trends of changes within the population. Conversely, the LCGA, a type of growth mixture modeling, which we employed for this study, is useful for identifying latent classes—subsets that differ in their characteristics, such as baseline values or rates of change—and examining the growth patterns among individuals included in these heterogeneous classes [49, 50]. There are several types of models that estimate the pattern of change, such as linear models, nonlinear models, and free estimation models. As this study aimed to estimate the trajectory of change at three time points over the course of 3 years, which is the minimum requirement for conducting a longitudinal study, we did not adopt a nonlinear model due to insufficient practical evidence to infer irregular changes at a certain time. In the case of the free parametric model, the parsimony of the model would be less due to the reduced degree of freedom [51]. Considering these points, the linear model was utilized to track the trajectory of change in our analysis.

In LCGA, the heterogeneous growth trajectories are identified through statistical model fit testing. The commonly used fit indices are the Akaike information criterion (AIC), Bayesian information criterion (BIC), sample-size adjusted Bayesian information criterion (SSABIC), entropy, and Vuong-Lo-Mendell-Rubin likelihood ratio test (VLMR). In general, a model is determined to have a good fit when the AIC, BIC, and SSABIC values are smaller, entropy is close to 1, and VLMR is significant. Moreover, models with classes that include fewer than 5% of the samples can be excluded [52]. These indices are sensitive to sample size. Therefore, the number of latent classes must be determined based on these criteria and in consideration of fit indices, the quality of classification, comparative analysis of models, study questions, and parsimony of interpretation of the identified latent classes [52, 53]. Finally, multinomial logistic regression analysis was performed to identify the predictors of the different types of changes in female drinking identified through LCGA.

## Results

### Participants’ general characteristics

Table 1 shows the general characteristics of the participants. In total, 47.6% of participants did not have a religion. Furthermore, 70.8% had a high school diploma or lower level of education, while 29.2% had a bachelor’s degree or higher. Moreover, 43.4% had a spouse and 51.5% had a job. The mean age was 46.7 years (SD: 9.93), and the mean natural log-converted income was 11.948 (SD: 0.26). The mean value of early depression was 3.749 (SD: 4.755) The mean level of problem drinking for women subjects was 1.013 (SD: 2.14) in the first year, 1.029 (SD: 2.05) in the second year, and .953 (SD: 2.03) in the third year.

**Table 1:** Participants’ general characteristics (*n* = 4615)

Variable	Category	N	%
**Religion**	No	2196	47.6
	Yes	2419	52.4
**Education**	≤ High school	3267	70.8
	University ≤	1348	29.2
**Spouse**	No	2001	43.4
	Yes	2614	56.6
**Job**	No	2239	48.8
	Yes	2376	51.5
		Mean	SD
**Age**		46.74	9.93
**(Ln)Income**		11.948	0.26
**Depression**		3.749	4.755
**Problem Drinking in Year 1**		1.013	2.14
**Problem Drinking in Year 2**		1.029	2.05
**Problem Drinking in Year 3**		.953	2.03

^a^Problem Drinking: Mean AUDIT value for all participants at each time point

### Classes of changes in female drinking identified through LCGA

Table 2 shows the models with a varying number of classes and the fit of each model. Of the models with low AIC, BIC, and SSABIC; entropy close to 1; significant VLMR; and at least 5% of the sample represented in each class, the three-class model had the best fit considering the parsimony of interpretation. Therefore, we chose the three-class model.

**Table 2:** Fit of latent class models for types of changes in female drinking

Class	Model fit					Classes				
						N(%)				
	AIC	BIC	SSABIC	Entropy	VLMR *P*-value	Class 1	Class 2	Class 3	Class 4	Class 5
2	−13,532.044	−13,461.236	− 13,496.190	.950	.001	4075 (88.3)	510 (11.7)			
**3**	**−15,302.547**	**−15,212.428**	**− 15,256.915**	**.962**	**.003**	**3708 (80.4)**	**670 (14.5)**	**237 (5.1)**		
4	−16,837.150	−16,727.720	−16,781.739	.986	.001	789 (17.1)	3406 (73.8)	347 (7.5)	73 (1.6)	
5	−16,996.685	−16,867.943	− 16,931.496	.954	.963	3490 (75.6)	562 (12.2)	210 (4.5)	156 (3.3)	197 (4.4)

^a^*AIC* Akaike information criterion, *BIC* Bayesian information criterion, *SSABIC* Sample-size adjusted Bayesian information criterion, *VLMR* Vuong-Lo-Mendell-Rubin likelihood ratio test

**Fig. 1:**
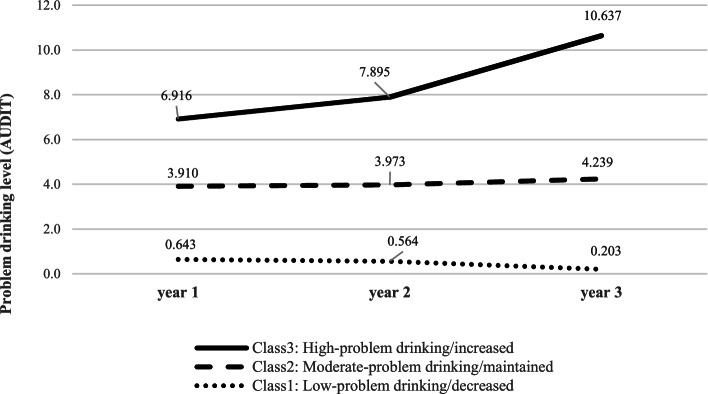
Trajectories of changes in the potential female classes for drinking in the final model

The types of changes in female drinking according to the final model are presented in Table 3 and Fig. 1. Each class was named based on the characteristics and according to the type of drinking change. The first class showed the lowest level of problem drinking at the baseline and a further decline in the level of problem drinking over time. Therefore, it was named the “low level/decreased” class. This class comprised 80.4% of the sample. The second class showed a moderate level of problem drinking at the baseline and only a slight increase in problem drinking over time and was named “moderate level/maintained” class. This class comprised 14.5% of the sample. The third class showed a higher baseline level of problem drinking and rapid escalation in problem drinking over time and was accordingly named “high level/increased” class. This class comprised 5.1% of the sample.

**Table 3:** Summary of trajectories of changes in the potential female classes for drinking in the final model

Potential classes	Potential class names	Rates to be in the potential class (N)	Trajectories of changes in problem drinking (Mean)		
			Year 1	Year 2	Year 3
Class 1	Low level/decreased	80.4 (3708)	.643	.564	.203
Class 2	Moderate level/maintained	14.5 (670)	3.910	3.973	4.239
Class 3	High level/increased	5.1 (237)	6.916	7.895	10.637

### Predictors of types of changes in female drinking

First, to check for multicollinearity among the major variables, a correlation analysis was performed. The correlation coefficients were below 0.8 and ranged from .007–.636, In addition, the Variance Inflation Factor (VIF) between the independent variables was calculated to be less than ten, with a minimum of 1.042 and a maximum of 6.924, indicating that multicollinearity was not present. Next, multinomial logistic regression was performed to identify the factors pertaining to gender-related experiences in the social structure that could predict the types of longitudinal changes in female drinking. The results are shown in Table 4. With reference to the “low level/decreased” class, the experience of domestic violence, work–family conflict, and perceived gender role were not statistically significant for the “moderate level/maintained” class. Meanwhile, the odds of being in the “moderate level/maintained” class were .750 times lower when patients had higher depression (Odds Ratio; OR = .750, *p* < .05) and 1.257 times higher when they had higher satisfaction with social relationships (OR = 1.257, *p* < .05). In terms of sociodemographic factors, the odds of being in the moderate level/maintained class, with reference to the low level/decreased class, were lower with advancing age (OR = .950, *p* < .001) and absence of religious beliefs (OR = .774, *p* < .01), and higher among the employed (OR = 1.746, *p* < .001). Further, the odds of being in the “high level/increased” class were 1.857 times higher with more experience of domestic violence (OR = 1.857, *p* < .05) and 1.309 times higher with higher levels of work–family conflict (OR = 1.309, *p* < .05). In terms of perceived gender roles, the odds of being in the “high level/increased” class, with reference to the “low level/decreased” class, were .705 times lower with more conservative gender role perceptions (OR = .705, *p* < .05). In terms of sociodemographic factors, the odds of being in the “high level/increased” class were lower with advancing age (OR = .932, *p* < .001), absence of religious beliefs (OR = .774, *p* < .01), and higher education level (OR = .607, *p* < .01), and lower among the employed (OR = 1.404, *p* < .05).

**Table 4:** Determinants of changes in alcohol consumption in women

	Low consumption/decreased (reference)			
	Moderate consumption/maintained		High consumption/increased	
	Exp(B)	95% CI	Exp(B)	95% CI
Age	.950^***^	.943 ~ .958	.932^***^	.920 ~ .943
Religion	.774^**^	.646 ~ .926	.601^**^	.449 ~ .806
Education	.822	.658 ~ 1.026	.607^**^	.435 ~ .848
Employment status	1.746^***^	1.448 ~ 2.106	1.404^*^	1.050 ~ 1.878
Spouse	.787	.500 ~ 1.239	.456	.234 ~ .888
Equivalized income	.972	.602 ~ 1.570	1.192	.581 ~ 2.445
Depression	.750^*^	.577 ~ .974	.668	.442 ~ 1.009
Satisfaction with social relationships	1.257^*^	1.053 ~ 1.502	1.291	.973 ~ 1.712
Experience of domestic violence	1.295	.897 ~ 1.869	1.857^*^	1.109 ~ 3.111
Work–family stress	1.132	.993 ~ 1.292	1.309^*^	1.068 ~ 1.604
Perceived gender roles	.925	.749 ~ 1.142	.705^*^	.511 ~ .973

†*p* < 0.1, *p < .05, **p < .01, ***p < .001

## Discussion

We examined the types of longitudinal changes in female drinking by analyzing 3 years of KOWEPS (2018–2020) data using LCGA and explored their features. We also identified the gender-related determinants of the different classes of changes in female drinking.

First, three classes emerged in relation to changes in drinking patterns among women: “low level/decreased,” “moderate level/maintained,” and “high level/increased.” This confirms that there were heterogeneous changes in drinking patterns among women. With reference to the cut-off criteria for AUDIT scores, the “moderate level/maintained” group could be considered to engage in social drinking. Further, the “high level/increased” group demonstrated hazardous drinking behaviors at the baseline, but progressed to suspected alcohol use disorder over time, suggesting that they are a more risky drinking group compared to the other groups. These are different from previous longitudinal studies, which used the traditional potential growth model, in which only the pattern of decrease or increase over time was considered depending on the predictor [10, 54, 55]. Potential classes that exhibited heterogeneous trends in the pattern of change regarding problem drinking among women were identified, which enabled the discovery of predictors that increased the likelihood of belonging to a class with undesirable problem drinking changes, providing evidence for early intervention strategies in problem drinking. Therefore, there is a need to tailor drinking prevention and intervention programs according to the type of change observed in female drinking behavior. For example, prevention programs for the “moderate level/maintained” group should be designed to help maintain their healthy use of alcohol, while programs for the “high level/increased group” should involve early screening and therapeutic interventions that seek to lower problem drinking.

Second, after adjusting for sociodemographic factors (e.g., age, religion) and psychosocial factors (e.g., depression, satisfaction with social relationships), which differ according to the types of changes in female drinking, we found: I) Gender-related factors were not significant for the “moderate level/maintained” class compared to other reference classes. However, depression and satisfaction with social relationships were significant predictors of drinking behavior. II) Unlike for the “moderate level/maintained” class, experience of domestic violence, work–family conflict, and perceived gender roles were identified as significant predictors in the “high level/increased” class. In other words, women with low levels of depression and high levels of satisfaction with social relationships were likely to be in the “moderate level/maintained” class, which engaged in a healthy and enjoyable level of drinking. Meanwhile, the odds of being in the “high level/increased class”—women engaging in hazardous drinking behaviors at the baseline and quickly progressing to suspected alcohol use disorder (addiction) over time—were higher among women with more experience of domestic violence, higher work–family conflict, and more open gender role perceptions. This supports previous findings that women victims of domestic violence frequently experience substance use disorder [56, 57], women often utilize drinking as a stress-coping strategy [58–60], and more conservative gender role perceptions are negatively correlated with drinking because women holding such views value family life more than their non-conservative counterparts do [9, 23, 36, 61].

In particular, it is noteworthy that, as per the traditional gender roles in Korean society, women are perceived to place more value on family and are required to fulfill their duties as a spouse, parent, daughter-in-law, and daughter, all of which contribute to controlling their alcohol consumption. This can also be understood in terms of the association between gender role perceptions and work–family conflict. Considering reports that women with more traditional gender role perceptions face greater difficulties in becoming economically active [47, 62], it can be speculated that women with more open gender role perceptions are relatively more economically active. Since socioeconomic activities increase the opportunities for alcohol consumption [17], gender role perceptions are likely to be closely associated with hazardous drinking [43]. Moreover, while women’s economic involvement is commonplace in modern society, failure to achieve an appropriate division of roles and household chores with their husbands can result in greater stress in women due to work–family conflict. Consequently, to relieve this stress, women may end up increasing their alcohol intake [17, 63].

From a gender perspective, the highest odds ratios, or the highest likelihood, of a woman being in the ‘high-problem drinking/increased class’, were associated with experiences of domestic violence, followed by work-family balance stress and perceived gender roles, in that order. Its implications are as follows: First, the experience of domestic violence is a strong predictor of being in the “high level/increased” group, and individuals experiencing post-traumatic stress disorder seek to lower alertness [64] or relieve negative emotions [65, 66] through continuous hazardous drinking. Therefore, it is important to assess drinking problems in female victims of domestic violence and provide services accordingly, to prevent their negative spiral into alcohol addiction.

Second, there is a need for programs in the community and workplaces to promote mental health or coping skills that can help women cope with the stress caused by their heavy role in socio-economic activities and housework. In modern society, the increase in participation from women in socio-economic activities is a natural phenomenon. However, women still shoulder the burden of housework and childcare, the stress of which can be the most prevalent cause of drinking behavior in them. Additionally, women with a high level of work-family stress are likely to experience increasing levels of problem drinking, not only due to existing social perceptions of women’s problem drinking, but also the fact that their commitment to treatment is limited by their primary role of family care. Therefore, when designing a problem drinking prevention policy or a drinking problem intervention program for women, it is necessary to consider measures such as the inclusion of a work-family stress reduction program.

Third, from the perspective of promoting women’s health, educational programs that improve social perceptions by separating women’s identity from gender role perceptions and programs that prevent problem drinking in women need to be developed. In terms of gender role attitudes (perceptions), society has traditionally been lenient toward male drinking, but relatively stricter toward female drinking [43]. However, increased economic participation of women [67] and improved awareness regarding gender equality and women’s rights [68] have diminished the social stigma and negative perceptions of female drinking. In line with these social changes, the mainstream industry and mass media in Korean society have been actively marketing to women [29, 69, 70]. Particularly, they strive to provide women who used to be constrained by gender norms and traditional roles with the image of women in modern society by linking drinking behavior with women, their right to self-determination, and freedom [70, 71]. In this regard, it is necessary to actively monitor alcohol marketing targeted at women and promote anti-alcohol campaigns and education in response to messages promoting drinking, thereby inducing healthy drinking habits and reducing harm.

Taken together, the alcohol consumption of women has increased dramatically in recent years. The combination of the perception that drinking liberates women who have been socially oppressed for a long time, and the implicit social coercion of drinking being essential for smooth work and social life contributes to patterns of problem drinking in women in South Korea. On the other hand, the problem of female drinking due to domestic violence is not a phenomenon that can only be explained by demographic, socioeconomic, and psychological factors. Therefore, this study emphasizes that problem drinking in women should be addressed from a gender perspective, in terms of their vulnerability within existing social and cultural hierarchies, based on their experiences. Gender-conscious policies should be proposed to reduce the harm of problem drinking in women, such as prevention education, improvement of the drinking environment, policy development, preparation of research and monitoring systems, and the establishment of leadership to implement them.

This study has several strengths. First, we shed light on the heterogeneous changes in drinking within the female population by not assuming that all women undergo the same changes in their drinking patterns over time. Second, we address the shortcomings of previous studies that have primarily examined sociodemographic, psychosocial, and relational factors as predictors of problem drinking in women. we analyze the effects of women’s gender-related experiences in the actual social structure on the longitudinal changes in their drinking patterns. This examination has enriched our discussion on female drinking from a gender perspective. However, the study is not without limitations. We could not examine gender gaps in the effects studied—we could not study how the gender perspective influences men. In addition, it was not possible to capture specific and minute changes of patterns by time point because we investigated patterns of change with data over 3 years, which is the minimum number of time points required for conducting a longitudinal study. Also, this study did not reflect the pattern of changes in independent variables, though they did exist. Finally, despite the change in social perceptions of women’s drinking, we are unable to rule out the possibility that respondents underreported their drinking due to pre-existing negative perceptions. Moreover, we believe that accounting for the limitations of our study and mapping gender differences and differences across generations (cohorts) within the female population would lead to more interesting findings.

## Conclusions

In recent years, the gender gap in the indices for alcohol use has narrowed, and alcohol consumption among women has increased. We identified three latent classes with different trajectories that reflect changing patterns in problem drinking. Furthermore, this study identified gender-specific predictors of the types of longitudinal changes in female drinking. Three classes of changes in female drinking emerged: “low problem drinking/decreased,” “moderate problem drinking/maintained,” and “high problem drinking/increased.” Gender-related factors in the social structure—experience of domestic violence, work–family conflict, and gender role perceptions—were identified as significant predictors of drinking only in the “high level/increased” group. These results highlight the need for interventions tailored to the specific classes of changes in female drinking patterns as opposed to a one-size-fits-all approach. They also show the necessity of formulating policies for reducing the harmful consequences of hazardous drinking in women by considering gender-related factors, such as domestic violence and work–family conflict.

## Data Availability

The dataset supporting the findings of this article is available upon reasonable request from the corresponding author.

## Abbreviations

KOWEPS: Korea Welfare Panel Study
LCGA: Latent class growth analysis
OR: Odds ratio
AUDIT: Alcohol use disorder identification test
ISSP: International Social Survey Programme
CES-D: Center for Epidemiologic Studies Depression Scale
AIC: Akaike information criterion
BIC: Bayesian information criterion
SSABIC: Sample-size adjusted Bayesian information criterion
VLMR: Vuong-Lo-Mendell-Rubin likelihood ratio test
